# Associations of body mass index and diabetes with hip fracture risk: a nationwide cohort study

**DOI:** 10.1186/s12889-018-6230-y

**Published:** 2018-11-29

**Authors:** Hsiu-Ling Huang, Cheng-Chin Pan, Yu-Fen Hsiao, Ming-Chih Chen, Chuan-Yu Kung, Pei-Tseng Kung, Wen-Chen Tsai

**Affiliations:** 1 Department of Social Work, Toko University, Chiayi County, Taiwan, Republic of China; 2 Department of Health Services Administration, China Medical University, 91, Hsueh-Shih Road, Taichung, 40402 Taiwan, Republic of China; 3 Department of Urology, Ministry of Health and Welfare, Hengchun Tourism Hospital, Hengchun, Taiwan, Republic of China; 4 Department of Orthopedics, Ministry of Health and Welfare, Hengchun Tourism Hospital, Hengchun, Taiwan, Republic of China; 5 Department of Nursing, Ministry of Health and Welfare, Hengchun Tourism Hospital, Hengchun, Taiwan, Republic of China; 6 Department of Healthcare Administration, Asia University, Taichung, Taiwan, Republic of China; 7 Department of Medical Research, China Medical University Hospital, China Medical University, Taichung, Taiwan, Republic of China

**Keywords:** BMI, Body mass index, Hip fracture, Diabetes, National Health Insurance

## Abstract

**Background:**

The high prevalence of diabetes is associated with body mass index (BMI), and diabetes can cause many complications, such as hip fractures. This study investigated the effects of BMI and diabetes on the risk of hip fractures and related factors.

**Methods:**

We retrospectively reviewed data from 22,048 subjects aged ≧ 40 years from the National Health Interview Survey in Taiwan (NHIST) in 2001, 2005, and 2009. We linked the NHIST data for individual participants with the National Health Insurance Research Database (NHIRD), which includes the incidence of hip fracture from 2000 to 2013. We defined five categories for BMI: low BMI (BMI < 18.5), normal BMI (18.5 ≦ BMI < 24), overweight (24 ≦ BMI < 27), mild obesity (27 ≦ BMI < 30), and moderate obesity (BMI ≧ 30). The Cox proportional hazards model was used to analyze the effects of BMI and diabetes on risk of hip fracture.

**Results:**

The Cox proportional hazards model shows that hip fracture risk in participants with diabetes was 1.64 times that of non-diabetes patients (95% confidence interval [CI]:1.30–2.15). Participants with low BMIs showed a higher hip fracture risk (HR: 1.75) than those with normal BMI. Among the five BMI groups, compared with non-diabetes patients, only diabetes patients with a normal BMI showed a significantly higher risk on hip fracture (HR: 2.13, 95% CI: 1.48–3.06). In participants with diabetes, compared with those with normal BMI, those with overweight or obesity showed significantly lower hip fracture risks (HR: 0.49 or 0.42). The hip fracture risk in participants who expend ≧ 500 kcal/week in exercise was 0.67 times lower than in those who did not exercise.

**Conclusions:**

Diabetes and low BMI separately are important risk factors for hip fracture. There was an interaction between diabetes and BMI in the relationship with hip fracture (*p* = 0.001). The addition of energy expenditure through exercise could effectively decrease hip fracture risk, regardless of whether the participants have diabetes or not. The results of this study could be used as a reference for health promotion measures for people with diabetes.

## Background

The worldwide incidence of hip fracture is predicted to increase from 1.66 million as of 1990 to 6.26 million by 2050 [1]. The global population is ageing, and hip fractures significantly affect the mobility and mortality of the elderly. The associated medical costs should also not be overlooked [2, 3]. Both type 1 and type 2 diabetes can increase hip fracture risk and complications due to abnormal bone metabolism [4, 5]. Vestergaard found that compared with those without diabetes, the relative risk (RR) for hip fractures in patients with type 1 diabetes was 6.94, and that for hip fractures in patients with type 2 diabetes was 1.38 [6]. In other studies, patients with longer diabetes duration were associated with a higher hip fracture risk compared to patients without diabetes. When the diabetes duration was < 5 years, RR was 1.8, but when the diabetes duration was > 15 years, RR increased to 2.66 [7, 8]. According to previous studies, diabetes was positively associated with risk of hip fracture.

The high prevalence of diabetes is related to population ageing and body mass index (BMI, unit: kg/m^2^). The World Health Organization (WHO) recommends using BMI as an important indicator of obesity [9]; the higher the BMI, the higher a patient’s risk is for metabolic diseases [10]. Being either overweight or obese can increase the incidence of type 2 diabetes, and the incidence of diabetes in obese adults is approximately 3–7 times that of adults with normal weight. The incidence in those with a BMI of > 35 is 20 times that in those with BMIs of 18.5–24.9 [11, 12].

To understand, a higher BMI level was associated with a higher prevalence of type 2 diabetes, and diabetes is an important risk facture for hip fracture. In addition to BMI and diabetes, the study from Søgaard et al. found that risk of hip fracture decreased with increasing BMI [13]. De Laet et al. [14] also found that people with a BMI of 30 kg/m^2^ showed a lower hip fracture risk (RR: 0.83; 95% confidence interval [CI]: 0.69–0.99) than those with a BMI of 25 kg/m^2^. BMI is associated with the incidence of fracture. Aurégan et al. [15] suggest that low BMI independently increase the risk of fractures. Johansson et al. [16] also found that low BMI was a risk factor for hip and all osteoporotic fracture. However, another study found that among postmenopausal women, obese women showed a higher risk of ankle and upper leg fractures than nonobese women [17]. Thus, low BMI is an important risk factor for fractures, but the relation between high BMI and fractures is not clear [16, 17].

Obesity is one of the major risk factors for type 2 diabetes, which may in turn also increase hip fracture risk. However, it is still uncertain whether BMI has an impact on hip fractures in diabetes patients. Thus, we investigated whether diabetes has same effects on risk of hip fracture in those with different BMI, and the effects of BMI on hip fracture risk in diabetes patients.

## Methods

### Data sources and participants

We retrospectively reviewed quadrennial data from the National Health Interview Survey in Taiwan (NHIST) for the years 2001, 2005, and 2009. The survey participants’ height and weight data were used to calculate the baseline BMI. We excluded pregnant women and participants younger than 40 years. We linked the NHIST participants’ individual data with the National Health Insurance Research Database (NHIRD), which includes nationwide data on all citizens in Taiwan. We extended the washout period to January 1st, 2000 for our participants in this study. All participants who had been diagnosed with hip fracture before NHIST survey were excluded from this study to make sure the temporal relationship between BMI/diabetes and hip fracture. We included a total of 22,048 participants and monitored hip fracture incidence for the period of 2000 to 2013.

The NHIST was conducted nationally and quadrennially by the Taiwan Health Promotion Administration. The information available from surveys include personal information, personal health status, knowledge about disease prevention, utilization of medical services, personal health behaviour, self-rated health status, and work and economic status, among others. The survey results provide a reference for developing and implementing healthcare policies in Taiwan [18].

This study was reviewed and approved by the research ethics committees of China Medical University (IRB No.: CMUH 103-REC3–109). We deleted all personal identification from the data analysed in this study to protect the patients’ personal identities. Taiwan’s National Health Insurance program was launched in March 1995, and as of 2013, the nationwide coverage rate was 99.68% [19]. This compulsory public health insurance program provides comprehensive information such as demographic data and data on all medical services, including prescription drugs, surgical treatments for outpatients, emergency care, and inpatient care. The NHIRD includes medical information on all citizens covered by insurance, including treatments for diabetes, hip fractures, and other conditions [20, 21]. The comprehensiveness and accuracy of the NHIRD have been confirmed by the Ministry of Health and Welfare, and the database has been used in numerous studies [22, 23].

### Variable descriptions

The variables examined were BMI, personal basic characteristics (sex, age), environmental factors (urban or rural residential areas), socio-economic status (monthly salary), health status (Charlson Comorbidity Index [CCI] and diabetes complication severity index [DCSI]), health behaviour (weekly energy expenditure through exercise), and diabetes status. The WHO has developed a classification of BMI for international use, but the index for overweight Asian adults is lower than the world average. Thus, many Asian countries have developed their own criteria for overweight and obesity. We used the BMI classification criteria of the Taiwan Health Promotion Administration and divided the participants into five categories: low BMI (BMI < 18.5), normal BMI (18.5 ≦ BMI < 24), overweight (24 ≦ BMI < 27), mild obesity (27 ≦ BMI < 30), and moderate obesity (BMI ≧ 30) [24].

In healthcare, diagnosis codes are used as a tool to group and identify diseases, disorders, symptoms, poisonings, adverse effects of drugs and chemicals, injuries, and other reasons for patient encounters. In the NHIRD, diagnosis codes are collected using the ICD-9-CM code (The International Classification of Diseases, Ninth Revision, Clinical Modification). We defined diabetes patients as those who received a diagnosis of diabetes (ICD-9-CM: 250) and at least three outpatient treatments or one hospitalization during the year of the interview survey or within 365 days before or after the survey [25]. We excluded patients with type 1 diabetes, gestational diabetes, neonatal diabetes, or impaired glucose tolerance (ICD-9-CM: 6488, 7751, 7902, 6480). We defined hip fracture as a diagnosis of femoral neck fracture, intertrochanteric fracture, or subtrochanteric fracture (ICD-9-CM: 820.XX) and having received one of the following surgical treatments: partial hip replacement (ICD-9-CM: 81.52), open reduction of fracture with internal fixation of the femur (ICD-9-CM: 79.35), or closed reduction of fracture with internal fixation of the femur (ICD-9-CM: 79.15).

We divided residential areas into seven levels from most urban and to least urban [26]. We calculated the severity of comorbidities based on the CCI revised by Deyo et al. [27] and divided them into groups with scores of 0, 1–3, and  ≧ 4. We calculated the DCSI based on seven types of diabetes complications (retinopathy, nephropathy, neuropathy, cerebrovascular, cardiovascular, peripheral vascular disease, and metabolic) as classified by Young et al [28] and used different weight scores (0 or  ≧ 1) to represent different severities.

In terms of health behaviour, we calculated the weekly energy expenditure through exercise according to the method proposed by Wen et al. [29] using the NHIST. Each type of exercise corresponds to a different Metabolic Equivalent of Task (MET, a unit of exercise intensity) according to the breathing status during exercise. One MET is defined as the oxygen uptake in ml/kg/min when sitting quietly (3.5 ml/kg/min). The weekly energy expended in exercise is calculated as follows: MET * frequency of exercises over the past 2 weeks (times) * each exercise duration (hours) * body weight (kg) * 7/14. We used the MET to collect and validate the weekly energy expenditure in kilocalories (kcal) for specific exercises, based on which participants were divided into three groups according to the expenditure per week: no exercise, < 500 kcal/week, and  ≧ 500 kcal/week.

### Statistical analysis

SAS statistical analysis software version 9.3 (SAS Institute, Cary, NC, U.S.A.) was used for the analysis, and *p*-values < 0.05 were considered statistically significant. In descriptive statistics, the participants’ variables were analysed, including BMI, diabetes status, basic personal characteristics (sex and age), environmental factors (urbanization degree of residential areas), social and economic status (monthly salary), health status (CCI and DCSI), and health behaviour (weekly energy expenditure through exercise). Our aim was to compare the numbers of subjects with hip fractures and percentage distributions. A chi-square test was used to perform analysis to determine the relationship between the variables and incidence of diabetes and level of BMI. A log-rank test was used to determine the relationship with hip fracture incidence.

For the inferential statistical analysis, a Cox proportional hazard model was used to investigate the effects of BMI and diabetes on hip fracture risk after controlling for variables such as personal basic characteristics, environmental factors, social and economic status, health status, and health behaviour. Further, in order to examine whether diabetes has a same effect on risk of hip fracture in those with different BMI, we also examined the interaction relationship between diabetes status and level of BMI on the risk of hip fracture. A stratified analysis was further performed to investigate the effects of diabetes on hip fracture risk in those with different BMI if there was an interaction relationship between diabetes status and level of BMI. Finally, the Cox proportional hazard model was used to investigate the effects of BMI on hip fracture incidence in participants with diabetes.

## Results

### Participant demographics and cox proportional hazard model analysis

A total of 22,048 subjects were eligible for inclusion in this study (Table 1), of which 3508 had diabetes and 315 had hip fractures. Among the different level of BMI groups, we found that the higher of the BMI, the higher prevalence of diabetes. When the participants had moderate obesity (BMI ≧ 30), diabetes risk was as high as 33.33%. There was significant difference between diabetes and non-diabetes patients in risk of hip fracture (*p* < 0.05). Additionally, participants with low BMI (BMI < 18.5) showed a higher hip fracture rate (3.56%) than other BMI subgroups. There were significant differences between participants with diabetes and those without diabetes in BMI, hip fracture, sex, age, urbanization of residence area, monthly salary, CCI, DCSI and weekly energy expenditure through exercise (*P* < 0 .05). In Table 2, there were significant differences in hip fracture incidence between the participants in terms of variables, including BMI, diabetes status, age, monthly salary, CCI, and DCSI (*p* < 0.05).

**Table 1:** Participant demographics with descriptive statistics

Variable	Total	%	Non-diabetes		Diabetes		*p-value*	BMI < 18.5		18.5≦BMI < 24		24 ≦ BMI < 27		27 ≦ BMI < 30		BMI≧30		*p-value*
			N	%	N	%		N	%	N	%	N	%	N	%	N	%	
Total	22,048	100.00	18,540	84.09	3508	15.91		786	3.56	10,858	49.25	6474	29.36	2742	12.44	1188	5.39	
BMI							< 0.001											
BMI < 18.5	786	3.56	711	3.83	75	2.14												
18.5 ≦ BMI < 24	10,858	49.25	9581	51.68	1277	36.40												
24 ≦ BMI < 27	6474	29.36	5358	28.90	1116	31.81												
27 ≦ BMI < 30	2742	12.44	2098	11.32	644	18.36												
BMI≧30	1188	5.39	792	4.27	396	11.29												
Diabetes																		< 0.001
No	18,540	84.09	–	–	–	–		711	90.46	9581	88.24	5358	82.76	2098	76.51	792	66.67	
Yes	3508	15.91	–	–	–	–		75	9.54	1277	11.76	1116	17.24	644	23.49	396	33.33	
Hip Fracture							< 0.001											< 0.001
No	21,733	98.57	18,308	98.75	3425	97.63		758	96.44	10,689	98.44	6396	98.80	2712	98.91	1178	99.16	
Yes	315	1.43	232	1.25	83	2.37		28	3.56	169	1.56	78	1.20	30	1.09	10	0.84	
Sex							0.036											< 0.001
Male	10,908	49.47	9230	49.78	1678	47.83		312	39.69	4944	45.53	3607	55.72	1474	53.76	571	48.06	
Female	11,140	50.53	9310	50.22	1830	52.17		474	60.31	5914	54.47	2867	44.28	1268	46.24	617	51.94	
Age							< 0.001											< 0.001
40–49	9015	40.89	8287	44.70	728	20.75		301	38.30	4728	43.54	2503	38.66	1039	37.89	444	37.37	
50–59	6347	28.79	5177	27.92	1170	33.35		176	22.39	2877	26.50	2031	31.37	871	31.77	392	33.00	
60–69	3616	16.40	2715	14.64	901	25.68		108	13.74	1652	15.21	1138	17.58	509	18.56	209	17.59	
70–79	2274	10.31	1740	9.39	534	15.22		119	15.14	1149	10.58	632	9.76	259	9.45	115	9.68	
≧80	796	3.61	621	3.35	175	4.99		82	10.43	452	4.16	170	2.63	64	2.33	28	2.36	
Urbanization of residence area							< 0.001											0.297
1 & 2	10,027	45.48	8524	45.98	1503	42.84		356	45.29	5016	46.20	2947	45.52	1180	43.03	528	44.44	
3 & 4	7234	32.81	6096	32.88	1138	32.44		255	32.44	3521	32.43	2122	32.78	940	34.28	396	33.33	
5–7	4787	21.71	3920	21.14	867	24.71		175	22.26	2321	21.38	1405	21.70	622	22.68	264	22.22	
Monthly salary (NTD)							< 0.001											< 0.001
≦ 17,280	2578	11.69	2373	12.80	205	5.84		136	17.30	1343	12.37	680	10.50	280	10.21	139	11.70	
17,281–22,800	9925	45.02	8149	43.95	1776	50.63		329	41.86	4864	44.80	2935	45.34	1254	45.73	543	45.71	
22,801–36,300	5121	23.23	4255	22.95	866	24.68		197	25.06	2459	22.65	1499	23.15	667	24.33	299	25.17	
> 36,300	4424	20.07	3763	20.30	661	18.84		124	15.78	2192	20.19	1360	21.01	541	19.73	207	17.42	
CCI							< 0.001											< 0.001
0	16,988	77.05	14,507	78.25	2481	70.72		550	69.97	8439	77.72	5042	77.88	2110	76.95	847	71.30	
1–3	4303	19.52	3376	18.21	927	26.43		189	24.05	2017	18.58	1240	19.15	563	20.53	294	24.75	
≧ 4	757	3.43	657	3.54	100	2.85		47	5.98	402	3.70	192	2.97	69	2.52	47	3.96	
DCSI							< 0.001											< 0.001
0	20,784	94.27	17,703	95.49	3081	87.83		737	93.77	10,300	94.86	6096	94.11	2565	93.54	1089	91.67	
≧ 1	1264	5.73	837	4.51	427	12.17		49	6.23	558	5.14	381	5.89	177	6.46	99	8.33	
Weekly energy expended of calories in exercise							< 0.001											< 0.001
No exercise	12,001	54.43	10,216	57.02	1785	51.49		464	64.27	5881	55.96	3434	54.25	1522	57.00	700	60.66	
< 500 kcal/week	3630	16.46	3045	16.99	585	16.87		142	19.67	1980	18.84	1029	16.26	345	12.92	134	11.61	
≧ 500 kcal/week	5755	26.10	4658	25.99	1097	31.64		116	16.07	2649	25.20	1867	29.49	803	30.07	320	27.73	
Missing	662	3.00																

*BMI* body mass index, *CCI* Charlson Comorbidity Index, *DCSI* diabetes complication severity index

*NTD* New Taiwan Dollar, 32 NTD = 1 US dollar

Urbanization of residence area (Level 1 was the most urbanized)

*p-value*: chi-square test

**Table 2:** Descriptive statistics of participants with or without hip fractures

Variable	Total	%	Without hip fractures		With hip fractures		*p* -value
			N	%	N	%	
Total	22,048	100.00	21,733	98.57	315	1.43	
BMI							< 0.001
BMI < 18.5	786	3.56	758	3.49	28	8.89	
18.5 ≦ BMI < 24	10,858	49.25	10,689	49.18	169	53.65	
24 ≦ BMI < 27	6474	29.36	6396	29.43	78	24.76	
27 ≦ BMI < 30	2742	12.44	2712	12.48	30	9.52	
≧ 30	1188	5.39	1178	5.42	10	3.17	
Diabetes							< 0.001
No	18,540	84.09	18,308	84.24	232	73.65	
Yes	3508	15.91	3425	15.76	83	26.35	
Sex							0.205
Male	10,908	49.47	10,762	49.52	146	46.35	
Female	11,140	50.53	10,971	50.48	169	53.65	
Age							< 0.001
40–49	9015	40.89	8993	41.38	22	6.98	
50–59	6347	28.79	6310	29.03	37	11.75	
60–69	3616	16.40	3547	16.32	69	21.90	
70–79	2274	10.31	2161	9.94	113	35.87	
≧ 80	796	3.61	722	3.32	74	23.49	
Urbanization of residence area							0.099
1 & 2	10,027	45.48	9903	45.57	124	39.37	
3 & 4	7234	32.81	7140	32.85	94	29.84	
5–7	4787	21.71	4690	21.58	97	30.79	
Monthly salary (NTD)							< 0.001
≦ 17,280	2578	11.69	2489	11.45	89	28.25	
17,281-22,800	9925	45.02	9793	45.06	132	41.90	
22,801-36,300	5121	23.23	5069	23.32	52	16.52	
> 36,300	4424	20.07	4382	20.16	42	13.33	
CCI							< 0.001
0	16,988	77.05	16,808	77.34	180	57.14	
1–3	4303	19.52	4197	19.32	105	33.33	
≧ 4	757	3.43	727	3.35	30	9.53	
DCSI							< 0.001
0	20,784	94.27	20,504	94.35	280	88.89	
≧ 1	1264	5.73	1229	5.65	35	11.11	
Weekly amount of calories burned in exercise							0.831
No exercise	12,001	54.43	11,845	56.09	156	57.99	
< 500 kcal	3630	16.46	3585	16.98	45	16.73	
≧ 500 kcal	5755	26.10	5687	26.93	68	25.28	
Missing	662	3.00					

*BMI* body mass index, *CCI* Charlson Comorbidity Index, *DCSI* diabetes complication severity index

*NTD* New Taiwan Dollar; 32 NTD = 1 US dollar

Urbanization of residence area (Level 1 was the most urbanized)

*p*-value: log-rank test

We also used the Cox proportional hazard model to analyze the effects of BMI and diabetes on hip fracture risk. The results of four models of are shown in Table 3. The first model is the univariate analysis of diabetes and hip fracture with unadjusted results, the second is for diabetes without BMI, the third is for BMI without the diabetes variable, and the final model is for both variables together. In the final model, we found that hip fracture risk in diabetes patients was 1.64 times the risk in non-diabetes patients (95% CI: 1.30–2.15, *p* < 0.05). Patients with low BMI showed a higher hip fracture risk (Adjusted hazards ratio [Adj. HR]: 1.75, 95% CI: 1.17–2.61) than those with normal BMI (reference). Additionally, patients who were overweight, mildly obese, or moderately obese had lower hip fracture risk than patients with normal BMI, but the differences were not statistically significant (*p* > 0.05). Hip fracture risk in female patients was 1.29 times that in male patients (95% CI: 1.03–1.62, *p* = 0.027). Compared with a reference group (aged 40–49 years), older patients showed a higher hip fracture risk (*p* < 0.05): among subjects  ≧ 80 years old, hip fracture risk was as high as 52.16 times the baseline risk. Separately, participants with higher CCI scores showed a higher hip fracture risk than the reference group (CCI = 0), and hip fracture risk in those who expended  ≧ 500 kcal/week in exercise was 0.67 times lower than in those who did not exercise (95% CI: 0.50–0.89).

**Table 3:** Cox proportional hazard model analysis of hip fracture risk in all participants

Variables	Unadjusted HR	*p-value*	Diabetes without BMI			BMI without diabetes			Diabetes and BMI together			
			Adjusted HR	95% CI		Adjusted HR	95% CI		Adjusted HR	95% CI		*p-value*
Diabetes												
No *(ref.)*												
Yes	2.04	< 0.001	1.54	1.18	2.00	–	–	–	1.64	1.30	2.15	< 0.001
BMI												
18.5 ≦ BMI < 24 *(ref.)*	–	–	–	–	–							
BMI < 18.5	2.58	< 0.001	–	–	–	1.71	1.14	2.56	1.75	1.17	2.61	0.007
24 ≦ BMI < 27	0.78	0.072	–	–	–	0.86	0.66	1.13	0.84	0.64	1.10	0.205
27 ≦ BMI < 30	0.72	0.098	–	–	–	0.75	0.51	1.11	0.70	0.48	1.04	0.076
BMI ≧ 30	0.60	0.111	–	–	–	0.61	0.32	1.16	0.55	0.29	1.05	0.072
Sex												
Male *(ref.)*												
Female	1.15	0.206	1.28	1.02	1.60	1.30	1.04	1.63	1.29	1.03	1.62	0.027
Age												
40–49 *(ref.)*												
50–59	2.66	0.000	2.47	1.45	4.19	2.63	1.55	4.46	2.50	1.47	4.25	0.001
60–69	9.12	< 0.001	7.81	4.79	12.72	8.62	5.31	14.01	7.87	4.83	12.83	< 0.001
70–79	27.67	< 0.001	23.19	14.41	37.33	24.86	15.47	39.94	22.68	14.08	36.52	< 0.001
≧ 80	75.60	< 0.001	56.01	33.99	92.29	58.24	35.39	95.87	52.16	31.60	86.09	< 0.001
Urbanization of residence area												
1 & 2 *(ref.)*												
3 & 4	0.96	0.787	0.97	0.74	1.28	0.96	0.73	1.26	0.96	0.73	1.26	0.768
5–7	1.28	0.073	1.21	0.91	1.60	1.17	0.88	1.56	1.18	0.89	1.57	0.251
Monthly salary (NTD)												
≦ 17,280 *(ref.)*												
17,281–22,800	0.36	< 0.001	0.71	0.52	0.97	0.78	0.57	1.05	0.70	0.52	0.96	0.025
22,801–36,300	0.34	< 0.001	0.85	0.58	1.23	0.93	0.64	1.35	0.84	0.58	1.22	0.349
> 36,300	0.27	< 0.001	0.87	0.58	1.31	0.98	0.66	1.45	0.86	0.58	1.29	0.471
CCI												
0 *(ref.)*												
1–3	2.55	< 0.001	1.58	1.23	2.04	1.56	1.22	2.01	1.58	1.23	2.04	0.000
≧ 4	5.44	< 0.001	2.58	1.73	3.86	2.46	1.65	3.66	2.64	1.77	3.95	< 0.001
DCSI												
0* (ref.)*												
≧ 1	2.55	< 0.001	1.34	0.93	1.93	1.43	0.99	2.07	1.35	0.93	1.95	0.112
Weekly energy expended of calories in exercise												
No exercise *(ref.)*												
< 500 kcal/week	0.78	0.129	0.80	0.57	1.11	0.81	0.58	1.13	0.80	0.57	1.11	0.181
≧ 500 kcal/week	0.75	0.038	0.65	0.49	0.86	0.68	0.51	0.91	0.67	0.50	0.89	0.006

*BMI* body mass index, *CCI* Charlson Comorbidity Index, *DCSI* diabetes complication severity index, *HR* hazard ratio, *CI* confidence interval

*NTD* New Taiwan Dollar; 32 NTD = 1 US dollar

Urbanization of residence area (overall 7 levels; Level 1 was the most urbanized)

We also tested the interaction relationship between diabetes status and level of BMI in risk of hip fracture. The result revealed that there was a significant interaction effect between diabetes status and level of BMI in hip fracture risk (*p* = 0.001).

### Stratified analysis of the effects of BMI and relative factors on hip fracture risk in diabetes patients

We used stratified analysis to examine the relative risk of hip fracture between diabetes and non-diabetes patients at different level of BMI (Table 4). After relevant variables were controlled in Cox proportional hazard model, hip fracture risk in diabetes patients was greater than that in non-diabetes patients regardless of BMI. Among the five BMI groups, compared with non-diabetes patients, only diabetes patients with a normal BMI (18.5 ≦ BMI < 24) showed a statistically significant difference in hip fracture risk (Adj. HR: 2.13, 95% CI: 1.48–3.06, *P* < 0.05). It means that diabetes increases risk of hip fracture but the magnitude of risk varies with the BMI level.

**Table 4:** Stratified analysis of the relative risk of hip fracture between diabetes and non-diabetes patients in terms of BMI

Variables	Diabetes patients			Non-diabetes patients			Adj. HR (diabetes vs. non-diabetic)		95% CI	*p* -value
	N	Hip fractures		N	Hip fractures (N) (%)					
		(N)	(%)		(N)	(%)				
Sum	3508	83	2.37	18,540	232	1.25	1.64	1.30	2.15	< 0.001
BMI										
BMI < 18.5	75	6	8.00	711	22	3.09	2.47	0.90	6.74	0.079
18.5 ≦ BMI < 24	1277	46	3.60	9581	123	1.28	2.13	1.48	3.06	< 0.001
24 ≦ BMI < 27	1116	17	1.52	5358	61	1.14	1.01	0.57	1.75	0.996
27 ≦ BMI < 30	644	9	1.40	2098	21	1.00	1.24	0.54	2.86	0.618
BMI ≧ 30	396	5	1.26	792	5	0.63	2.37	0.57	9.84	0.236

Note: Cox proportional hazards model was used and controlled for sex, age, urbanization of residence area, monthly salary, CCI, DCSI and weekly energy expenditure through exercise

### Analysis of the effects of relative factors on hip fracture risk in diabetes patients

**Fig. 1:**
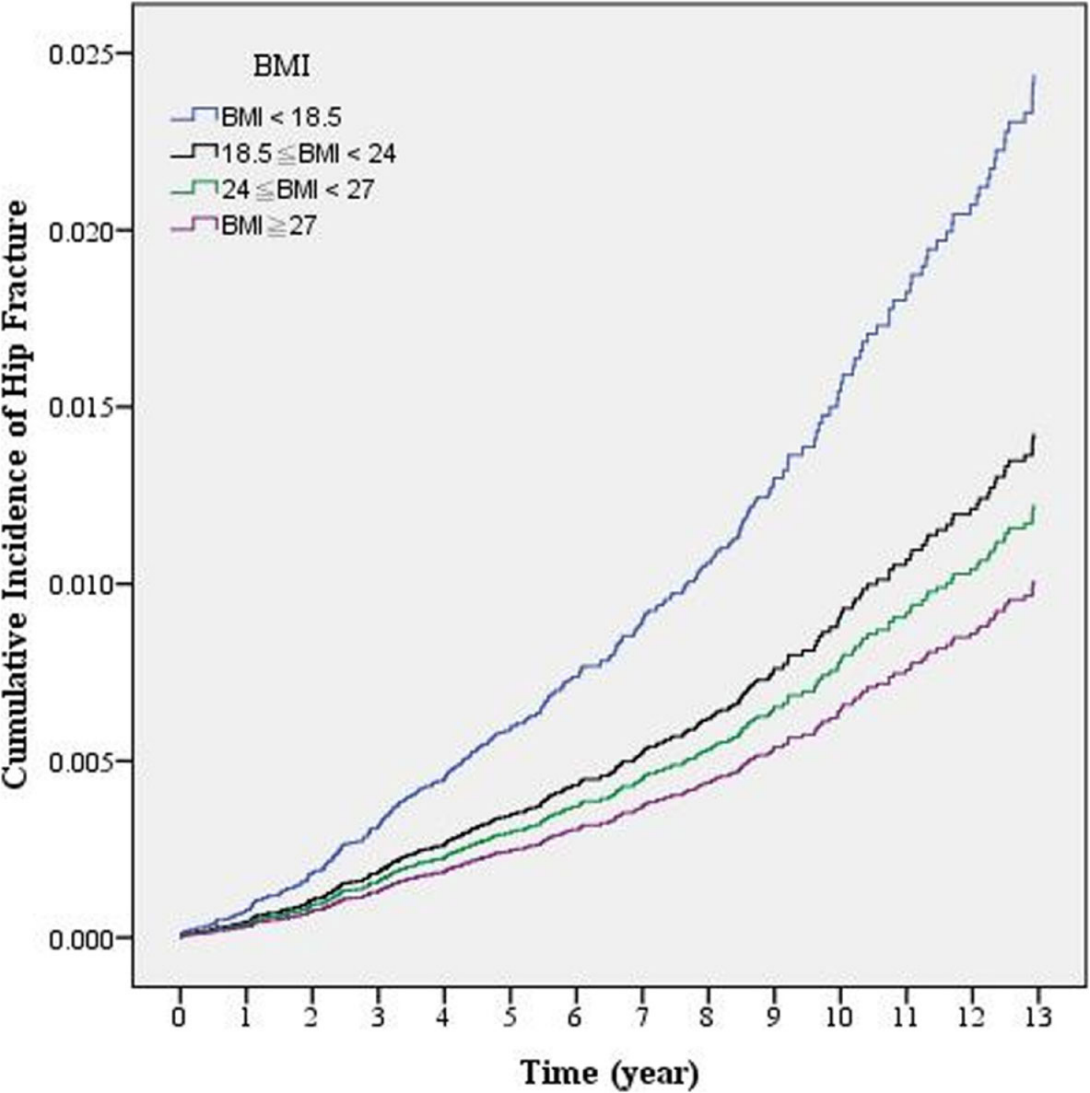
Comparisons of hip fracture risk among different BMI groups in patients with diabetes (After controlling for sex, age, urbanization of residence area, monthly salary, CCI, DCSI and weekly energy expenditure through exercise)

A Cox proportional hazard model was used to analyze diabetes patients (Table 5 & Fig. 1). Compared with a reference group (normal BMI, 18.5 ≦ BMI < 24), those with overweight (24 ≦ BMI < 27) or obesity (BMI ≧ 27) showed a lower hip fracture risk (Adj. HR: 0.49 vs. 0.42, *p* < 0.05). Compared with the reference group (aged 40–49 years), older patients showed a higher hip fracture risk, but statistically significant differences were only observed in patients  ≧ 60 years old (*p* < 0.05). Among diabetes patients, those with higher CCI or DCSI scores were associated with a higher hip fracture risk. As the weekly energy expended in exercise increased in diabetes patients, hip fracture risk decreased compared with diabetes patients without exercise. In particular, when the weekly energy expenditure was  ≧ 500 kcal/week, hip fracture risk in diabetes patients was significantly decreased to 0.54 times (95% CI: 0.31–0.94, *p* < 0.05).

**Table 5:** Analysis of the effect of BMI on hip fracture risk in diabetes patients

Variables	Unadjusted HR	*p-value*	Adjusted HR	95% CI		*p-value*
BMI						
18.5 ≦ BMI < 24 *(ref.)*						
BMI < 18.5	2.54	0.032	1.78	0.75	4.26	0.193
24 ≦ BMI < 27	0.42	0.002	0.49	0.28	0.85	0.012
BMI ≧ 27	0.38	0.001	0.42	0.23	0.78	0.006
Sex						
Male *(ref.)*						
Female	1.34	0.189	1.33	0.85	2.08	0.215
Age						
40–49 *(ref.)*						
50–59	1.81	0.258	1.73	0.62	4.88	0.298
60–69	5.16	0.001	4.67	1.78	12.25	0.002
70–79	11.32	< 0.001	10.20	3.82	27.27	< 0.001
≧ 80	23.02	< 0.001	16.97	5.94	48.43	< 0.001
Urbanization of residence area						
1 & 2 *(ref.)*						
3 & 4	0.76	0.289	0.79	0.46	1.36	0.402
5–7	0.81	0.425	0.84	0.47	1.47	0.535
Monthly salary (NTD)						
≦ 17,280 *(ref.)*						
17,281–22,800	0.55	0.122	0.74	0.34	1.63	0.457
22,801–36,300	0.78	0.548	1.09	0.47	2.51	0.837
> 36,300	0.42	0.058	0.63	0.25	1.61	0.338
CCI						
0 *(ref.)*						
1–3	2.17	0.001	1.51	0.92	2.47	0.106
≧ 4	5.63	< 0.001	3.51	1.43	8.59	0.006
DCSI						
0 *(ref.)*						
≧ 1	2.70	< 0.001	1.68	0.94	3.03	0.082
Weekly energy expended of calories in exercise						
No exercise *(ref.)*						
< 500 kcal/week	0.73	0.314	0.65	0.35	1.22	0.178
≧ 500 kcal/week	0.59	0.050	0.54	0.31	0.94	0.029

*BMI* body mass index, *CCI* Charlson Comorbidity Index, *DCSI* diabetes complication severity index;

*HR* hazard ratio, *CI* confidence interval

*NTD* New Taiwan Dollar, 32 NTD = 1 US dollar

Urbanization of residence area (overall 7 levels; Level 1 was the most urbanized)

## Discussion

This study is the first to use nationwide survey data in combination with data from the NHIRD to investigate the effects of BMI, diabetes, and relative factors on hip fracture risk. According to findings from previous studies, multiple complex factors in diabetes patients may cause abnormal bone metabolism and increase fracture incidence and subsequent complications [5, 6]. After controlling for other variables (including BMI), we also found that hip fracture risk in patients with type 2 diabetes was 1.64 times that in non-diabetes patients (Table 3), which was consistent with previous studies [4, 5, 30].

The analysis results in Table 3 show that all participants with low BMI (< 18.5) had a higher hip fracture risk (Adj. HR: 1.75, 95% CI: 1.17–2.61, *p* = 0.007) than those with normal BMI (18.5 ≦ BMI < 24). De Laet et al. [14] used a meta-analysis approach to study nearly 60,000 men and women from 12 cohorts of both Asian and Western participants. They found that low BMI conferred a significant risk for all types of fractures in both Asian and Western populations. They found that low BMI is an important risk factor for hip fractures. There were similar findings in the diabetes patients group (Table 5), but the result was not significant (Adj. HR: 1.78, 95% CI: 0.75–4.26, *p* = 0.193). After further analysis, only 83 diabetes patients had hip fractures. In addition, it was found that only six individuals had hip fractures among 75 diabetes patients with low BMI (BMI < 18.5). We believe that if the number of subjects was increased or if the subjects were observed for a longer period of time, the statistical results in variables could perhaps become significant.

To understand whether hip fracture risk of diabetes was the same in patients with different BMI, stratified analysis was performed (Table 4). Analysis results showed that diabetes patients had a higher hip fracture risk than non-diabetes patients in all MBI subgroups, but only those with a normal BMI showed significant differences (Adj. HR: 2.13, 95% CI: 1.48–3.06). It reflected the impact of diabetes on risk of hip fracture were not constant in people with different BMIs. The effect of diabetes on increasing hip fracture risk was more significant in those with lower BMI. The results indicated that diabetes as a risk factor for hip fracture was not independent of BMI, which was a novel finding.

Many studies have pointed out that overweight and obesity can increase the incidence of metabolic diseases [11, 12]. The similar result was also found in Table 1. Participant with a higher BMI had higher risks in type 2 diabetes. However, as shown in Table 5 and Fig. 1, we found that diabetes patients with high BMI (24 ≦ BMI < 27) or obesity (BMI ≧ 27) showed a lower hip fracture risk (Adj. HR: 0.49 vs. 0.42, *p* < 0.05) compared with the reference group (normal BMI). This is consistent with the report by Johansson et al [16], who analysed > 300,000 women from more than 25 countries and found that 87% of hip fractures occurred in those without obesity (defined as BMI ≧ 30 kg/m^2^). Furthermore, a relatively high BMI decreased the fracture risk in these women. The same result was found in all participants in which high BMI was protective against hip fracture, but there was no significant difference from the reference group (normal BMI, Table 3).However, high BMI is hardly a public health strategy that should be advocated, given concerns about cardiovascular disease in this population.

### Limitations

There were several limitations to our analyses. Data from the NHIRD were used for the analysis, so not all health behaviours and other factors were included in the analysis, such as eating habits, body composition/muscle mass, muscle function/sarcopenia, and history of falls history. Moreover, the duration of diabetes in all subjects and their blood glucose control status were not known.

## Conclusion

In this study, we found that diabetes increased hip fracture risk (HR: 1.64), and both diabetes and BMI had an interaction on risk of hip fracture (*P* = 0.001). The findings from this study revealed the following: (1) those with diabetes sustain more hip fractures, (2) low BMI was a risk factor for hip fracture, (3) The effect of diabetes on increasing hip fracture risk was more significant in those with lower BMI, and (4) physical exercise was important in preventing hip fractures, including among patients with diabetes.

It was not even clear whether any exercise was a significant protective factor in individuals with diabetes alone, or whether the result was driven by the general population. However, we found that energy expenditure through exercise  ≧ 500 kcal/week per week could effectively decrease hip fracture risk in the general population and in those with diabetes. Regardless of BMI or diabetes status, exercise helps prevent falls and hip fractures and was therefore especially important for diabetes patients. Hence, health education for diabetes on managing body weight and increasing the amount of exercise could effectively prevent hip fractures. The results of this study could be used as a reference for health education and health promotion measures for diabetes patients.

## Abbreviations

Adj. HR: Adjusted hazards ratio
BMI: Body mass index
CCI: Charlson Comorbidity Index
CI: Confidence interval
DCSI: Diabetes complication severity index
ICD-9-CM: The International Classification of Diseases, Ninth Revision, Clinical Modification
MET: Metabolic Equivalent of Task
NHIRD: National Health Insurance Research Database
NHIST: National Health Interview Survey in Taiwan
NTD: New Taiwan Dollar
RR: Relative risk
US: United States
WHO: World Health Organization
